# Vaginal Progesterone for Pregnancy Prolongation After Arrested Preterm Labor: A Randomized Clinical Trial

**DOI:** 10.1001/jamanetworkopen.2024.19894

**Published:** 2024-07-08

**Authors:** Zohar Nachum, Yael Ganor Paz, Manal Massalha, Malak Wated, Noa Harel, Enav Yefet

**Affiliations:** 1 Department of Obstetrics and Gynecology, Emek Medical Center, Afula, Israel; 2 Ruth and Bruce Rappaport Faculty of Medicine, Technion–Israel Institute of Technology, Haifa, Israel; 3 Department of Obstetrics and Gynecology, Wolfson Medical Center, Holon, Israel; 4 Department of Obstetrics and Gynecology, Samson Assuta Ashdod University Hospital, Ashdod, Israel; 5 Faculty of Medicine, Tel Aviv University, Tel Aviv, Israel; 6 Department of Obstetrics and Gynecology, Tzafon Medical Center, Poriya, Israel; 7 Azrieli Faculty of Medicine, Bar-Ilan University, Safed, Israel

## Abstract

**Importance:**

Women with arrested preterm labor (APTL) are at very high risk for spontaneous preterm delivery (SPTD), the leading cause of neonatal mortality and morbidity. To date, no maintenance therapy has been found to be effective for pregnancy prolongation. A few clinical trials with considerable methodological limitations have demonstrated some efficacy for 400 mg vaginal micronized progesterone (VMP) in women with APTL.

**Objective:**

To investigate the effectiveness of daily 400 mg VMP for the prolongation of pregnancy after APTL.

**Design, Setting, and Participants:**

This randomized clinical trial was conducted between December 19, 2018, and February 27, 2023, in 3 university-affiliated medical centers in Israel. Participants included women with singleton and twin pregnancies after APTL following tocolysis at 24 weeks 0 days to 34 weeks 0 days’ gestation. Women with a history of preterm delivery or asymptomatic cervical shortening in the current pregnancy were excluded.

**Interventions:**

Participants were randomly allocated to receive VMP 200 mg twice a day or no treatment until 36 weeks 6 days’ gestation.

**Main Outcomes and Measures:**

The primary end points were mean number of days from study enrollment to delivery and the rate of SPTD prior to 37 weeks’ gestation.

**Results:**

A total of 129 participants were enrolled (65 in the VMP group and 64 in the no-treatment group). Mean (SD) age was 27.6 (5.1) years. Between the VMP and no-treatment groups, there was no difference in pregnancy prolongation (mean [SD], 40.0 [17.8] vs 37.4 [20.3] days; *P* = .44) and the rate of SPTD (16 [25%] vs 19 [30%]; relative risk, 0.8; 95% CI, 0.5-1.5; *P* = .52). In twin pregnancies, including 12 and 15 pairs in the VMP and no-treatment groups, respectively, VMP prolonged pregnancy (mean [SD], 43.7 [18.1] vs 26.1 [15.2] days; *P* = .02), postponed the delivery week (36.5 [1.4] vs 34.7 [2.2] weeks; *P* = .01), shortened the length of stay in the neonatal intensive care unit (4.9 [10.6] vs 13.2 [18.5] days; *P* = .03) and overall hospital stay (8.3 [9.6] vs 15.1 [17.2] days; *P* = .03), and was associated with a higher birth weight (2444 [528] vs 2018 [430] g; *P* = .01).

**Conclusions and Relevance:**

These findings show that VMP given in a dosage of 200 mg twice a day following APTL is not an effective treatment to prolong pregnancy or prevent SPTD. However, VMP demonstrated beneficial effects in twin pregnancies, warranting further investigation.

**Trial Registration:**

ClinicalTrials.gov Identifier: NCT02430233

## Key Points

**Question:**

Is vaginal micronized progesterone (VMP) at 200 mg twice a day effective for pregnancy prolongation after arrested preterm labor?

**Findings:**

In this randomized clinical trial in 129 women with arrested preterm labor between 24 weeks 0 days and 34 weeks 0 days’ gestation, VMP did not prolong pregnancy or prevent spontaneous preterm delivery compared with no treatment. In a subgroup of 27 pairs of twins, VMP significantly prolonged pregnancy from a mean (SD) 26 (15) to 44 (18) days.

**Meaning:**

These findings suggest that after arrested preterm labor, VMP in a dosage of 200 mg twice a day is not useful as a maintenance therapy in singleton pregnancies but warrants further investigation in twin pregnancies.

## Teaser

This randomized clinical trial investigates the effectiveness of vaginal micronized progesterone for the prolongation of pregnancy after arrested preterm labor.

## Introduction

Preterm delivery (PTD) is the leading cause of neonatal mortality and morbidity.^1^ Since prematurity complications could be devastating, once preterm labor (PTL) is diagnosed before 34 weeks’ gestation, a tocolytic treatment is initiated in an effort to prolong the pregnancy.^2^ Several randomized clinical trials (RCTs) have failed to prove effectiveness of tocolytic treatment, such as nifedipine,^3,4^ β-adrenergic agonists,^5^ oxytocin antagonists,^6^ and magnesium sulfate,^7^ as maintenance therapies. Since women with arrested PTL (APTL) are at the highest risk for PTD, a great deal of effort has been invested in the search for a useful agent to prolong their pregnancies.

Previously, progestins have been suggested to be useful in preventing spontaneous PTD (SPTD) in women with cervical shortening or previous SPTD.^8,9,10,11,12,13^ In a meta-analysis evaluating the effectiveness of progestins, cerclage, and pessary to prevent SPTD in singleton at-risk pregnancies, vaginal progesterone was found to be the only intervention with consistent effectiveness.^14^ Two meta-analyses of RCTs that evaluated the effect of vaginal progesterone for pregnancy prolongation after APTL showed conflicting results, with the authors concluding that the findings did not support vaginal progesterone use following APTL.^15,16^ Most of the analyzed studies used 200 mg vaginal progesterone. Interestingly, 3 of 4 RCTs that used 400 mg vaginal progesterone demonstrated significant pregnancy prolongation in the vaginal progesterone group,^12,17,18,19^ yet those studies had considerable methodological limitations. In addition, the effect of vaginal micronized progesterone (VMP) in twin pregnancies was not investigated.^15,16^ In the present study, we investigated the effect of daily 400 mg VMP for the prolongation of pregnancy after APTL in singleton and twin pregnancies.

## Methods

This multicenter RCT was conducted between December 19, 2018, and February 27, 2023, at 3 Israeli university teaching medical centers (Emek, Tzafon, and Assuta Ashdod). The study protocol (Supplement 1) was authorized by the local review boards. Participants provided written informed consent. The study followed the Consolidated Standards of Reporting Trials (CONSORT) reporting guideline.

Women aged 18 years or older with PTL arrested by tocolytic treatment comprised the study population. Each woman underwent vaginal examination once before tocolysis initiation and once before study enrollment. Preterm labor was defined as at least 3 uterine contractions each lasting 30 seconds or longer per 30 minutes, as confirmed by external tocography and the presence of 1 of the following: (1) short cervix (≤25 mm) or (2) cervical dilatation of 1 to 4 cm accompanied by cervical effacement of 50% or greater.^20,21,22^

Cervical length was measured when the cervix was closed. The use of cervical length was based on departmental protocol according to studies that demonstrated its efficacy to predict SPTD in symptomatic women.^23,24^ When the cervix was dilated, cervical length measurement was not mandatory, since cervical dilatation and effacement were used to define PTL.

Gestational age at initiation of tocolytic treatment was between 24 weeks 0 days and 34 weeks 0 days. The tocolytic treatment, which included nifedipine, atosiban, or indomethacin, was determined by the attending physician at the time of PTL diagnosis and subsequently according to departmental protocol. All patients received betamethasone for fetal lung maturity and appropriate group B *Streptococcus* prophylaxis if indicated. The tocolytic treatment successful in arresting the PTL was continued until 48 hours from the first betamethasone injection.

Arrest of PTL was defined as a stable condition without progression to active labor, ie, 6 or fewer contractions per hour, intact membranes, and 4 cm or less cervical dilation after at least 24 hours from tocolytic initiation. Women were enrolled 24 hours after tocolytic initiation and up to 3 days after completing the tocolytic treatment.

We excluded women with suspected chorioamnionitis, significant placental abruption, intrauterine fetal death, major fetal malformations, allergy to progesterone, current use of progesterone, epilepsy, breast cancer, preterm premature rupture of membranes (PPROM), active liver disease, history of deep vein thrombosis, major active psychiatric disorders, uncontrolled chronic hypertension, heart failure, chronic kidney failure, pregestational diabetes with known target organ damage, and previous tocolytic treatment during the current pregnancy. We also excluded women with asymptomatic cervical shortening or previous SPTD, since VMP was indicated in those women anyway.

Participants were randomly assigned 1:1 to the study groups using a computer randomization sequence generation program with a block size of 4. Randomization by gestational age was stratified to 24 weeks 0 days to 28 weeks 6 days and 29 weeks 0 days to 34 weeks 0 days according to the day of tocolytic initiation. The randomization code was stored in sealed opaque envelopes in a closed study box until the intervention was assigned by the study physicians.

### Interventions

Patients diagnosed with APTL were allocated to receive either VMP, 200 mg twice a day (Utrogestan; Besins Healthcare) vs no treatment. Both participants and physicians were unmasked to group allocation. The first dose of the VMP was administered at least 24 hours after initial treatment with tocolytics and up to 3 days after completing the tocolytic treatment until 36 weeks 6 days’ gestation or delivery (for those delivering before 36 weeks 6 days). The cervical length by ultrasonography was documented prior to group allocation. In cases of PPROM, the VMP treatment was discontinued without participant removal from the study.

Ongoing monitoring included routine obstetric care and antepartum testing per the treating physician. Participants were followed up every 3 to 5 weeks, with maternal adverse effects documented and patients’ compliance assessed. Compliance was assessed by self-reporting of the number of tablets taken in the VMP group and ensuring that participants in the no treatment group did not take progestins. Final visits occurred at 36 to 37 weeks’ gestation or were conducted by telephone if the participant preferred. One repeated course of betamethasone and tocolytics was given in repeated PTL at least 1 week after the first course and up to 34 weeks 0 days’ gestation.

### Study End Points

The primary end points were the mean number of days from enrollment to delivery and the rate of SPTD (due to spontaneous labor or PTD following induction or cesarean delivery due to PPROM prior to 37 weeks). Secondary end points included pregnancy prolongation until 37 weeks (ie, the number of days until the 37th gestational week). We addressed this end point since it was the period when the participants received the study treatment and the most important time to prevent birth. Additional secondary end points were the number of days from recruitment to repeated PTL episode or PPROM up to 37 weeks, pregnancy prolongation beyond 1 week, need for repeated tocolysis, number of hospitalizations and length of stay until 36 weeks 6 days’ gestation, and the rate of SPTD (defined as spontaneous labor or PPROM prior to 37 weeks). The rate of chorioamnionitis and endometritis, postpartum hemorrhage, manual exploration of uterus and cervix, urinary tract or vulvovaginal infection until 36 weeks 6 days’ gestation and adverse medication reactions were collected. Neonatal outcomes included admission to the neonatal intensive care unit (NICU); length of NICU stay; length of hospital stay; fetal or neonatal death; birth weight; the rate of small for gestational age neonates (defined as <10th percentile of the neonatal Israeli birth weight curve^25^); and the rate of neonatal complications (as defined by the neonatologists), including transient tachypnea, respiratory distress syndrome, chronic lung disease, ventilatory support, supplemental oxygen, intraventricular hemorrhage, necrotizing enterocolitis, patent ductus arteriosus, retinopathy, neonatal sepsis, and congenital abnormalities.

### Statistical Analysis

Assuming a mean (SD) difference between the groups of 7 (14) days, a sample size of 64 participants per group was required (2-sided α of 5%, 80% power). This sample size was sufficient to detect a reduction from 50% to 25% in the rate of participants with SPTD (2-sided α of 5%; 84% power).

We set 80% power to be the minimal power of the primary end points. We calculated a sample size with 80% power to the mean difference hypothesis of the primary end point that required the largest sample size. Then, we calculated the power of the hypothesis of the second primary end point given the calculated sample size. We chose the mean (SD) difference of 7 (14) days based on previous studies of progesterone treatment following APTL that were summarized in a meta-analysis,^16^ which demonstrated a mean difference of 9 days between the progesterone and control groups and an SD range of 10 to 35 days. A mean (SD) difference of 7 (14) days is both a conservative and clinically significant assumption. The second end point was based on previous studies that demonstrated a rate of 50% of PTD following APTL^16^ and a reduction of 50% in the rate of PTDs following VMP administration to high-risk populations.^14^

The analysis was done according to the intention-to-treat principle. Intercohort baseline characteristics and outcomes were compared using Student *t* test (or Wilcoxon 2-sample test) for continuous variables and χ^2^ test (or Fisher exact test) for categorical variables. Adjustment for multiplicity of the primary end points was made using the Holm method.^26^ Interactions between variables were assessed using 2-way analysis of variance or multivariable logistic regression.

We evaluated the time in days from enrollment to delivery up to 37 weeks using the Kaplan-Meier method. A log-rank test was performed to compare the groups’ survival curves. Finally, we performed a prespecified subanalysis of the study end points following study recruitment between 24 weeks 0 days and 28 weeks 6 days and 29 weeks 0 days or longer and exploratory subanalyses of the study end points in singleton and twin pregnancies according to parity, time of tocolytic treatment initiation, cervical dilatation at enrollment, and greater than 80% compliance, defined as taking at least 80% of the tablets in the VMP group and not taking progesterone in the no-treatment group. We also performed a per-protocol analysis in which we compared participants who took at least 80% of the VMP tablets with the rest of the participants (who took fewer tablets or none at all).

Statistical analyses were performed using SAS, version 9.4 software (SAS Institute, Inc). Significance was set at a 2-sided *P* < .05.

## Results

**Figure 1.**
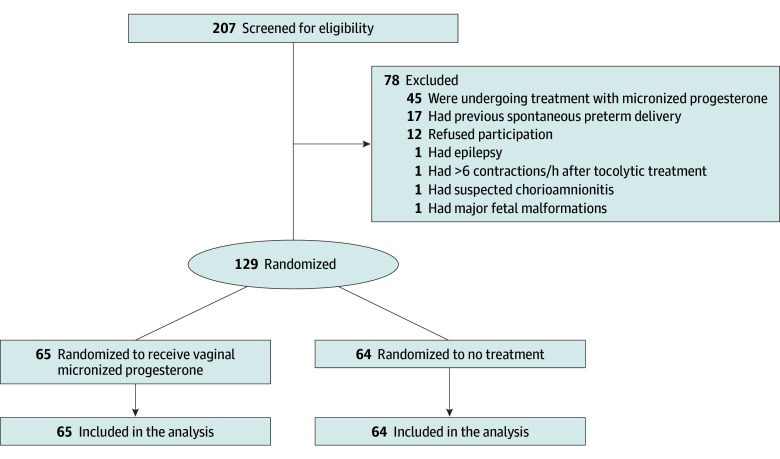
Patient Flow Diagram

Among the 207 women screened for eligibility to participate in this study, 129 participated (Figure 1). Mean (SD) age was 27.6 (5.1) years. Participants were allocated to receive VMP (n = 65) or no treatment (n = 64). Among those in the VMP group, 52 of 64 (81%) participants took more than 80% of the tablets (51 took all the tablets; 1 took 82% of the tablets). In the no-treatment group, no participant took progestins; thus, overall compliance was 90%. Participants’ characteristics were comparable between the groups (Table 1). The study included 12 and 15 pairs of twins in the VMP and no-treatment groups, respectively. Overall, 30 neonates (39%) were female and 47 (61%) male in the VMP group, and 27 neonates (34%) were female and 52 (66%) male in the no-treatment group. The study end points are presented in Table 2. There was no difference between the VMP and no-treatment groups in the mean number of days from enrollment to delivery (mean [SD], 40.0 [17.8] vs 37.4 [20.3] days; *P* = .44), the rate of SPTD (16 participants [25%] vs 19 participants [30%]; relative risk, 0.8; 95% CI, 0.5-1.5; *P* = .52]), and the overall rate of PTDs (19 [28%] vs 28 [44%]; relative risk, 0.7; 95% CI, 0.4-1.1; *P* = .09). Other end points also were not different between the groups.

**Table 1.** Maternal and Pregnancy Characteristics

Characteristic^a^	No. of participants (%)	
	VMP (n = 65)	No treatment (n = 64)
Maternal age, mean (SD), y	27.4 (5.0)	27.9 (5.2)
BMI before pregnancy, mean (SD)	23.2 (3.9)	23.8 (5.0)
No. of previous deliveries	0.7 (1.0)	0.8 (1.3)
No. of previous early miscarriages	0.3 (0.7)	0.2 (0.6)
No. of previous late miscarriages	0	0
Multiple pregnancies	12 (18)	15 (23)
Urinary tract infection during pregnancy	11 (17)	8 (13)
Pregestational diabetes	1 (1.5)	0
Gestational diabetes	3 (5)	8 (13)
Gestational week of tocolytic treatment	31.4 (2.3)	31.2 (2.1)
Tocolytic medication		
Nifedipine	65 (100)	64 (100)
Atosiban	25 (38)	19 (30)
Indomethacin	2 (3)	3 (5)
Gestational wk at recruitment, mean (SD)	31.7 (2.3)	31.6 (2.1)
Cervical length,^b^ mean (SD), mm		
Before tocolytic treatment	18 (6)	18 (5)
Before recruitment	21 (8)	22 (9)
Cervical dilatation before tocolytic treatment, mean (SD), cm	1.2 (0.8)	1.2 (0.7)
Cervical effacement before tocolytic treatment, %	70 (20)	60 (20)
Presenting part station before tocolytic treatment, mean (SD)	−2.4 (0.6)	−2.3 (0.6)
Cervical dilatation before recruitment, mean (SD), cm	0.8 (0.7)	0.9 (0.8)
Cervical effacement before recruitment, %	50 (20)	50 (20)
Presenting part station before recruitment, mean (SD)	−2.6 (0.5)	−2.4 (0.7)

Abbreviations: BMI, body mass index (calculated as weight in kilograms divided by height in meters squared); VMP, vaginal micronized progesterone.

^a^ Missing data for no-treatment group: BMI, 2 participants; cervical length before tocolytic treatment, 24 participants; cervical length before recruitment, 5 participants; cervical effacement before tocolytic treatment, 1 participant; cervical effacement before recruitment, 1 participant. Missing data for VMP group: cervical length before tocolytic treatment, 28 participants; cervical length before recruitment, 3 participants.

^b^ Cervical length was used to evaluate preterm labor only in closed cervix; therefore, this measurement was not mandatory in all women.

**Table 2.** Maternal and Neonatal End Points

End point	Entire cohort				Twin pregnancies			
	VMP, No. (%) or mean (SD)	No treatment, No. (%) or mean (SD)	*P* value	RR (95% CI)	VMP, No. (%) or mean (SD)	No treatment, No. (%) or mean (SD)	*P* value	RR (95% CI)
**Maternal end points**								
No. of participants	65	64	NA	NA	12	15	NA	NA
Gestational wk at delivery	37.4 (2.8)	36.9 (2.4)	.28	NA	36.5 (1.4)	34.7 (2.2)	.01	NA
Pregnancy prolongation since recruitment, mean (SD), d	40.0 (17.8)	37.4 (20.3)	.44	NA	43.7 (18.1)	26.1 (15.2)	.02	NA
Pregnancy prolongation until 37 wk, d	30.9 (14.7)	31.2 (16.5)	.93	NA	42.0 (17.6)	25.9 (15.0)	.03	NA
Pregnancy prolongation >7 d	62 (95)	59 (92)	.49	1.03 (0.9-1.1)	12 (100)	13 (87)	.49	1.2 (1.0-1.4)
Overall PTD	19 (29)	28 (44)	.09	0.7 (0.4-1.1)	5 (42)	12 (80)	.06	0.5 (0.25-1.1)
SPTD	16 (25)	19 (30)	.52	0.8 (0.5-1.5)	3 (25)	6 (40)	.68	0.6 (0.2-2.0)
Need for additional tocolytic treatment	3 (5)	5 (8)	.49	0.6 (0.1-2.4)	0	1 (6.7)	>.99	NA
PPROM	13 (20)	9 (14)	.37	1.4 (0.7-3.1)	1 (8.3)	3 (20)	.61	0.4 (0.05-3.5)
No. of hospitalizations until 37 wk	0.6 (0.8)	0.6 (0.7)	.89	NA	1.1 (0.9)	0.8 (0.8)	.45	NA
Length of hospital stay until 37 wk, d	1.9 (3.7)	1.8 (2.6)	.79	NA	4.2 (6.8)	2.9 (4.1)	.52	NA
No. of PTL events	0.2 (0.4)	0.3 (0.4)	.19	NA	0.2 (0.4)	0.4 (0.5)	.21	NA
No. of UTI events until 37 wk	0.2 (0.4)	0.3 (0.8)	.53	NA	0.1 (0.3)	0.1 (0.3)	.91	NA
No. of VVI events until 37 wk	0.03 (0.2)	0.02 (0.1)	.58	NA	0.1 (0.3)	0	.30	NA
Delivery mode								
Vaginal	49 (77)	45 (71)	.76	NA	7 (29)	7 (25)	.72	NA
Vacuum extraction delivery	2 (3)	4 (6)			2 (8)	1 (4)		
Cesarean delivery	14 (22)	14 (22)			15 (63)	20 (71)		
Labor induction	19 (30)	17 (27)	.74	1.1 (0.6-1.9)	7 (58)	5 (36)	.25	1.6 (0.7-3.8)
Chorioamnionitis^a^	6 (9)	2 (3)	.27	3 (0.6-14.1)	0	0	NA	NA
Endometritis	1 (2)	0	>.99	NA	0	0	NA	NA
Postpartum hemorrhage	7 (11)	3 (5)	.32	2.3 (0.6-8.5)	1 (8)	1 (7)	>.99	1.3 (0.1-18.0)
Manual exploration of the uterine cavity and cervix	6 (9)	2 (3)	.27	3 (0.6-14.1)	1 (8)	1 (7)	>.99	1.3 (0.1-18.0)
**Neonatal end points**								
No. of neonates	77	79	NA	NA	24	30	NA	NA
Neonatal sex								
Female	30 (39)	27 (34)	.54	1.1 (0.8-1.7)	12 (50)	14 (47)	.81	1.1 (0.6-1.9)
Male	47 (61)	52 (66)			12 (50)	16 (53)		
Birth weight, g	2762 (650)	2641 (661)	.25	NA	2444 (528)	2018 (430)	.01	NA
SGA	5 (6)	3 (4)	.49	1.7 (0.4-6.9)	3 (13)	1 (3)	.31	3.7 (0.4-34.0)
Apgar score at 1 min	9 (0.9)	8.9 (1.1)	.90	NA	8.7 (1.1)	8.9 (1)	.51	NA
Apgar score at 5 min	9.8 (0.5)	9.8 (0.6)	.28	NA	9.7 (0.6)	9.6 (0.7)	.67	NA
Cord pH	7.3 (0.1)	7.3 (0.1)	.22	NA	7.3 (0.1)	7.3 (0.1)	.42	NA
Length of hospital stay, d	7.2 (11.6)	8.7 (12.2)	.22	NA	8.3 (9.6)	15.1 (17.2)	.03	NA
NICU admission	15 (19)	23 (29)	.16	0.7 (0.4-1.2)	7 (29)	17 (57)	.04	0.5 (0.3-1.0)
Length of NICU stay, d	5 (13.4)	6.1 (13.3)	.19	NA	4.9 (10.6)	13.2 (18.5)	.03	NA
Fetal or neonatal death	0	0	NA	NA	0	0	NA	NA
Transient tachypnea	7 (9)	3 (4)	.21	2.4 (0.6-8.9)	2 (8)	3 (10)	>.99	0.8 (0.2-4.6)
Respiratory distress syndrome	2 (3)	5 (6)	.44	0.4 (0.1-2.1)	1 (4)	4 (13)	.37	0.3 (0.0-2.6)
Chronic lung disease	1 (1)	1 (1)	>.99	1.03 (0.1-16.1)	0	1 (3)	>.99	NA
Ventilator support	8 (10)	7 (9)	.75	1.2 (0.4-3.1)	2 (8)	6 (20)	.28	0.4 (0.1-1.9)
Supplemental oxygen	10 (13)	8 (10)	.58	1.3 (0.5-3.1)	4 (17)	6 (20)	>.99	0.8 (0.3-2.6)
Intraventricular hemorrhage	1 (1)	2 (3)	>.99	0.5 (0.1-5.5)	1 (4)	2 (7)	>.99	0.6 (0.1-6.5)
Necrotizing enterocolitis	0	0	NA	NA	0	0	NA	NA
Patent ductus arteriosus	1 (1)	0	.49	NA	0	0	NA	NA
Retinopathy	1 (1)	0	.49	NA	0	0	NA	NA
Neonatal sepsis	1 (1)	0	.49	NA	0	0	NA	NA
Congenital abnormalities not previously identified	3 (4)	3 (4)	>.99	1.03 (0.2-4.9)	0	3 (10)	.25	NA

Abbreviations: NA, not applicable; NICU, neonatal intensive care unit; PPROM, preterm premature rupture of membranes; PTD, preterm delivery; PTL, preterm labor; RR, relative risk; SGA, small for gestational age; SPTD, spontaneous preterm delivery; UTI, urinary tract infection; VMP, vaginal micronized progesterone; VVI, vulvovaginal infection.

^a^ Four and 2 participants in the VMP and no-treatment groups, respectively, had subclinically positive culture findings for placental or membrane growth. The rest had clinical chorioamnionitis.

The study characteristics and end points were compared separately for singleton (eTables 1 and 2 in Supplement 2) and twin (eTable 3 in Supplement 2; Table 2) pregnancies. The study characteristics and end points were comparable in singleton pregnancies in the VMP and no-treatment groups.

In twin pregnancies, maternal and pregnancy characteristics were similar between the groups (eTable 3 in Supplement 2). Time from enrollment to delivery was longer in the VMP group than in the no-treatment group overall (mean [SD], 43.7 [18.1] vs 26.1 [15.2] days; *P* = .02) and until 37 weeks (mean [SD], 42.0 [17.6] vs 25.9 [15.0] days; *P* = .03), and delivery week was later (mean [SD], 36.5 [1.4] vs 34.7 [2.2] weeks; *P* = .01). Additionally, the VMP group had more favorable neonatal outcomes compared with the no-treatment group, including shorter length of NICU stay (mean [SD], 4.9 [10.6] vs 13.2 [18.5] days; *P* = .03), shorter overall length of hospital stay (mean [SD], 8.3 [9.6] vs 15.1 [17.2] days; *P* = .03), and a higher birth weight (mean [SD], 2444 [528] vs 2018 [430] g; *P* = .01) (Table 2).

**Figure 2.**
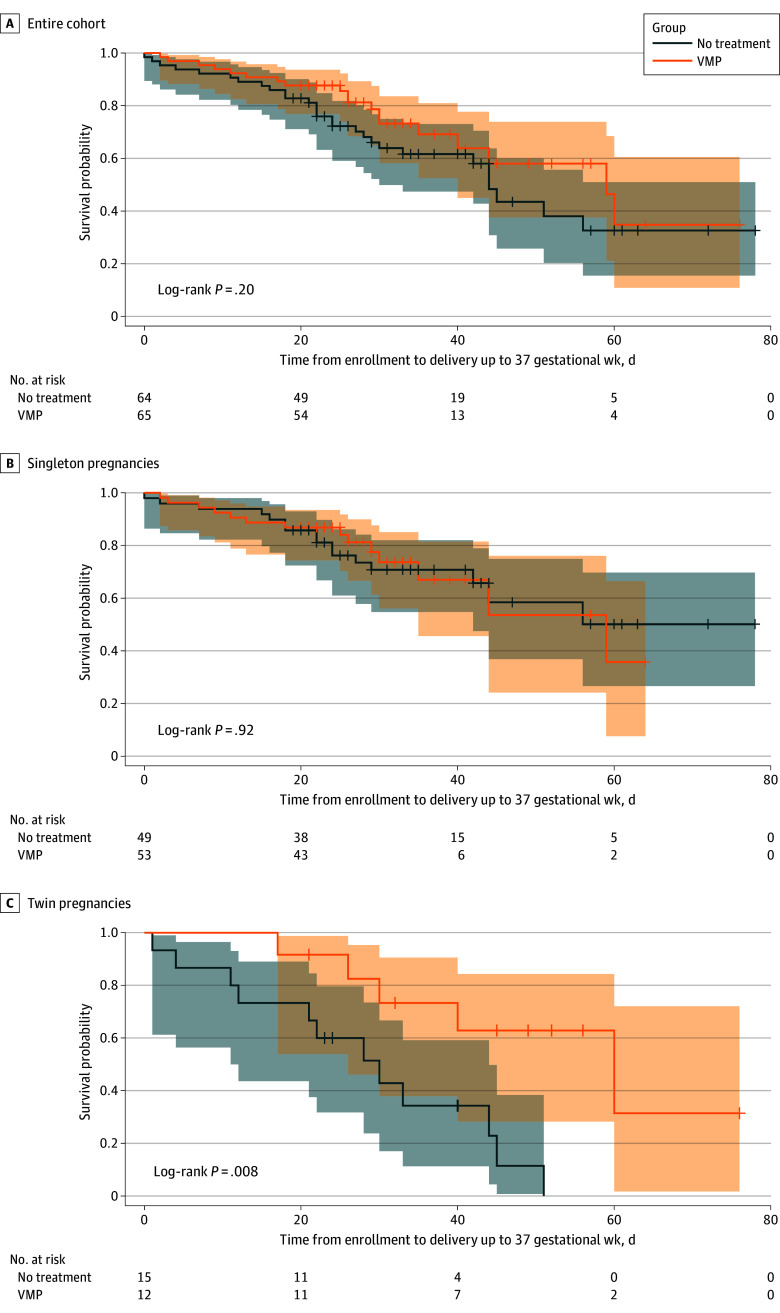
Kaplan-Meier Survival Analysis of Time From Enrollment to Delivery Up to 37 Gestational Weeks Tic marks indicate censored data; shaded areas indicate 95% CIs. VMP indicates vaginal micronized progesterone.

Kaplan-Meier survival curves of the time from enrollment to delivery up to 37 weeks in the VMP and no-treatment groups demonstrated similar times to event for the entire cohort and singleton pregnancies. In twin pregnancies, the time to event was longer for the VMP group (Figure 2). We further analyzed whether multiple gestation modified the effect of VMP on the study outcomes by assessing the interaction between the study treatment and multiple gestation. The interaction terms were statistically significant for time from enrollment to delivery and until 37 weeks, for length of NICU stay and overall length of hospital stay, and for birth weight. The interaction term for delivery week and the rate of NICU admission was not significant.

We performed several subanalyses of the study end points (Table 3). No parameter was found to affect the study end points evaluated. We also performed a per-protocol analysis and compared those end points between participants who took at least 80% of the VMP tablets (n = 52) and the rest of the participants (n = 77). We performed a power calculation according to the per-protocol numbers, which remained 80%. The study end points were also not different in this comparison .

**Table 3.** Subanalyses According to Various Characteristics

Characteristic	No. of total participants (%)		*P* value
	VMP	No treatment	
**Recruitment**			
24 wk 0 d to 28 wk 6 d gestation			
Pregnancy prolongation >7 d	10 of 10 (100)	11 of 11 (100)	>.99
Overall preterm delivery	6 of 10 (60)	5 of 11 (45)	.67
Spontaneous preterm delivery	6 of 10 (60)	4 of 11 (36)	.39
Pregnancy prolongation from recruitment, mean (SD), d	54 (27.1)	61 (23.3)	.60
Pregnancy prolongation until 37 weeks, mean (SD), d	48.2 (21.3)	54.5 (17.4)	.67
≥29 wk 0 d gestation			
Pregnancy prolongation >7 d	52 of 55 (95)	48 of 53 (91)	.48
Overall preterm delivery	13 of 55 (24)	23 of 53 (43)	.03
Spontaneous preterm delivery	10 of 55 (18)	15 of 53 (28)	.21
Pregnancy prolongation from recruitment, mean (SD), d	37.4 (14.5)	32.5 (15.9)	.09
Pregnancy prolongation until 37 wk, mean (SD), d	27.8 (10.8)	26.4 (11.6)	.50
**Parity**			
Primiparous			
Pregnancy prolongation >7 d	35 of 38 (92)	34 of 37 (92)	>.99
Overall preterm delivery	12 of 38 (32)	15 of 37 (41)	.42
Spontaneous preterm delivery	11 of 38 (29)	10 of 37 (27)	.85
Pregnancy prolongation from recruitment, mean (SD), d	40.5 (19.9)	36.8 (18.8)	.41
Pregnancy prolongation until 37 wk, mean (SD), d	31 (16.5)	31.1 (15.3)	.97
Multiparous			
Pregnancy prolongation >7 d	27 of 27 (100)	25 of 27 (93)	.49
Overall preterm delivery	7 of 27 (26)	13 of 27 (48)	.09
Spontaneous preterm delivery	5 of 27 (19)	9 of 27 (33)	.21
Pregnancy prolongation from recruitment, mean (SD), d	39.2 (14.6)	38.2 (22.5)	.56
Pregnancy prolongation until 37 wk, mean (SD), d	30.9 (12.1)	31.3 (18.4)	.74
**Initiation from tocolytic treatment**			
>48 h			
Pregnancy prolongation >7 d	42 of 44 (95)	59 of 64 (92)	.70
Overall preterm delivery	13 of 44 (30)	28 of 64 (44)	.14
Spontaneous preterm delivery	11 of 44 (25)	19 of 64 (30)	.59
Pregnancy prolongation from recruitment, mean (SD), d	39.7 (17.5)	37.4 (20.3)	.54
Pregnancy prolongation until 37 wk, mean (SD), d	30.4 (14.5)	31.2 (16.5)	.79
≤48 h			
Pregnancy prolongation >7 d	21 of 22 (95)	59 of 64 (92)	>.99
Overall preterm delivery	6 of 22 (27)	28 of 64 (44)	.17
Spontaneous preterm delivery	5 of 22 (23)	19 of 64 (30)	.53
Pregnancy prolongation from recruitment, mean (SD), d	40.7 (18.3)	37.4 (20.3)	.50
Pregnancy prolongation until 37 wk, mean (SD), d	31.7 (15.3)	31.2 (16.5)	.73
**Cervical dilation**			
None			
Pregnancy prolongation >7 d	28 of 30 (93)	28 of 29 (97)	>.99
Overall preterm delivery	7 of 30 (23)	8 of 29 (28)	.71
Spontaneous preterm delivery	7 of 30 (23)	6 of 29 (21)	.81
Pregnancy prolongation from recruitment, mean (SD), d	43.1 (20.3)	45.2 (19)	.68
Pregnancy prolongation until 37 wk, mean (SD), d	31.6 (17.1)	38.3 (16.6)	.14
≥1 cm			
Pregnancy prolongation >7 d	34 of 35 (97)	31 of 35 (89)	.36
Overall preterm delivery	12 of 35 (34)	20 of 35 (57)	.054
Spontaneous preterm delivery	9 of 35 (26)	13 of 35 (37)	.30
Pregnancy prolongation from recruitment, mean (SD), d	37.3 (15.1)	30.9 (19.2)	.13
Pregnancy prolongation until 37 wk, mean (SD), d	30.3 (12.5)	25.3 (14.2)	.12
**Compliance** ^a^			
>80%			
Pregnancy prolongation >7 d	50 of 52 (96)	59 of 64 (92)	.46
Overall preterm delivery	15 of 52 (29)	28 of 64 (44)	.10
Spontaneous preterm delivery	12 of 52 (23)	19 of 64 (30)	.42
Pregnancy prolongation from recruitment, mean (SD), d	40.3 (18.8)	37.4 (20.3)	.43
Pregnancy prolongation until 37 wk, mean (SD), d	32.1 (15.6)	40.3 (18.8)	.75

Abbreviation: VMP, vaginal micronized progesterone.

^a^ Compliance was defined as taking at least 80% of the progesterone tablets in the VMP group and not taking progesterone tablets in the no-treatment group.

Among participants who took VMP tablets (n = 57), 4 (7%) reported adverse effects. One participant reported abdominal pain and 1 reported diarrhea, and they discontinued treatment after 14 and 4 days, respectively. One participant reported nausea and vomiting and 1 reported vaginal burning sensation without treatment discontinuation.

## Discussion

In this RCT, we investigated the effect of VMP, 200 mg twice a day for the prolongation of pregnancy after APTL in singleton and twin pregnancies. When tested on the entire cohort or in singleton pregnancies, VMP did not prolong pregnancy or decrease the rate of SPTDs compared with no treatment. Other maternal and neonatal end points also were not affected. In contrast, in twin pregnancies, VMP prolonged pregnancy, shortened the length of NICU and overall hospital stay, and was associated with a higher birth weight. We chose as the primary end point the time from enrollment to delivery because we wanted to evaluate the effect of VMP treatment in prolonging pregnancy. We chose the same time period in the no-treatment group to maintain uniformity. In addition, gestational week of tocolytic treatment and gestational week at recruitment were not different between the groups, and in a subanalysis of treatment initiation from tocolytic treatment up to 48 hours or more, the results were similar.

The literature shows conflicted findings with regard to the efficacy of VMP for pregnancy prolongation after APTL. Two recent meta-analyses assessed the effectiveness of progesterone derivatives for pregnancy prolongation after an episode of APTL in singletons.^15,16^ One assessed the effectiveness of intramuscular 17α-hydroxyprogesterone caproate and oral, vaginal, or rectal natural or micronized progesterone in 13 RCTs (1722 women). Progestogen maintenance therapy was associated with a longer latency time of 4 days, and neonates were 124 g heavier compared with control neonates. However, when analyzing studies with low risk of bias only or studies that used vaginal progesterone (5 RCTs in 609 women), those effects were not demonstrated.^15^ The other meta-analysis tested specifically for the effect of vaginal progesterone in 13 RCTs (1658 women). Risk of PTD was similar in women who received vaginal progesterone and those in the control group. Pregnancy prolongation was 9 days longer among women who received vaginal progesterone, but this effect was only demonstrated in the subgroup of studies that were not placebo controlled. The authors concluded that the data did not support the use of vaginal progesterone in these settings.^16^

The current study was designed to overcome several methodological limitations of previous studies. First, we did not use magnesium sulfate for tocolysis. In many studies, magnesium sulfate was used as a tocolytic treatment, which has been shown to be no better than placebo to arrest PTL,^27,28^ and therefore, it is questionable whether the population in those studies indeed included women with APTL. Second, we collected data on twin pregnancies, as there were limited data regarding the effect of VMP or any other progesterone derivatives on twin pregnancies. In a meta-analysis, only 1 study included 4 pairs of twins in the progesterone and placebo groups each, but the study was terminated early, and this number was too small to make any conclusion regarding twin pregnancies.^16^ Finally, most studies that used vaginal progesterone did not show efficacy when a dose of 200 mg was administered.^15,16^ Only 4 studies used 400 mg of VMP once daily, all of them from a single country (Iran).^12,17,18,19^ Three of the studies demonstrated efficacy in pregnancy prolongation,^12,17,19^ but they had substantial methodological limitations, including small sample size, a considerable rate of loss to follow-up, use of magnesium sulfate as the tocolytic treatment, termination of treatment at 34 weeks, and unclear masking.^15^ In addition, in those studies, VMP was given once daily as opposed to our study in which 400 mg was divided into 2 doses. In a study that evaluated serum progesterone levels after a single dose of 200 mg VMP in 18 to 23 weeks’ gestation, the median time to peak was 12 hours, with a return to baseline after 24 hours.^29^ Therefore, administration of VMP in divided doses every 12 hours better corresponds with its pharmacokinetic properties.

In the current study, VMP prolonged pregnancy and reduced unfavorable neonatal outcomes in twin pregnancies. Although the number of twins in this study was small, to our knowledge, it is larger than in previous studies that evaluated the effect of progesterone to prolong pregnancy after APTL. Administration of vaginal progesterone to asymptomatic women with a twin gestation and a short cervix on ultrasonography in the second trimester reduced the risk of PTD when a dose of 400 mg, but not lower doses, was given.^30,31^ This observation is consistent with our findings. On the contrary, other studies that evaluated the effect of progesterone to prevent PTD in unselected populations of twin pregnancies^32,33^ and women with twins and a prior singleton SPTD^34^ did not show benefit.

It is unclear why the effect of VMP was demonstrated in twin pregnancies and not in singleton pregnancies. The difference may be related to the mechanism of action of progesterone derivatives and the pathophysiology of PTL in twin pregnancies, which is different than PTL of singleton pregnancies. Comparing PTDs of singletons vs twins, placentas from PTDs of singletons were characterized by a higher rate of maternal and fetal inflammatory responses, retroplacental hemorrhage, and vascular lesions related to maternal malperfusion. In addition, a higher rate of neonatal sepsis was observed in the singleton PTDs compared with the twin PTDs.^35^ In another study that compared myometrial contractility in singleton vs twin pregnancies using biopsy samples obtained from women not experiencing labor but undergoing cesarean delivery, the frequency of contractions and responses to oxytocin were found to be significantly increased in the twin pregnancies. In addition, the contractile activity correlated with increasing levels of stretch (using neonatal birth weights as a surrogate for uterine stretch), with response to oxytocin being significantly positively correlated with birth weight.^36^ Progesterone derivatives exert their action by reducing uterine contractility via suppression of myometrial activation, reduced expression of myometrial gap junctions, and contraction-related proteins.^37,38^ Therefore, it may be useful to prevent SPTD when the underlying pathophysiology is related to the overdistended uterus and increased contractility that characterize PTL of twin pregnancies and may be less effective in inflammation, infections, and vascular lesions, which are more prevalent in PTL of singleton pregnancies. An alternative explanation is that the risk for SPTD is higher in twin pregnancies to begin with, and therefore, the beneficial effects of VMP may become evident. This risk is relevant especially if twin pregnancy is combined with placental dysfunction where the higher the risk of preeclampsia in the first trimester, the earlier gestational age at birth with no preeclampsia.^39^ That progesterone may reduce the risk of spontaneous birth in twin pregnancies of less than 32 weeks only in women with a cervical length of less than 30 mm may also support this claim.^32^

Our results may lay the cornerstone for future studies on the effects of PTD prevention in twin pregnancies after APTL. Future studies with larger sample sizes should also examine the effect of VMP on perinatal morbidity and mortality.

In this study, 47 neonates (61%) of the VMP group and 52 (66%) of the no-treatment group were male. Male fetal sex has been found to be a risk factor for SPTD, the reason for which is unclear.^40^

### Strengths and Limitations

The strengths of this study are its multicenter RCT design, high rate of recruitment, and adherence to the study protocol. Additional strengths are the use of 2 primary outcomes to assess possible benefits of VMP for pregnancy prolongation and the performance of subanalyses. Several aspects of the study design strengthen the findings, including the inclusion of twin pregnancies, the use of evidence-based effective tocolytic treatments, and the use of VMP, 200 mg twice a day.

This study also has several limitations. First, there was a small number of twin pregnancies, and analyses within this group were not included as the primary end point. Furthermore, the inclusion of both singleton and twin pregnancies increased the heterogeneity of the study population. Second, high-risk populations for PTD, ie, women with a previous PTD or short cervix in the current pregnancy, were not included because, in our departments, VMP is indicated in those women and allocation to the control group would cause ethical issues. Third, inclusion of multiparous women without previous PTD also increased the heterogeneity of the study, yet in the subanalysis of primiparous and multiparous women, the study end points were similar and did not show a beneficial effect of VMP in either group. Fourth, the study was not powered to assess neonatal outcomes, multiple subanalyses, and the open-label design. Since the outcomes of this study were pregnancy prolongation and PTD, the open-label design may not have influenced the results. Finally, allocation concealment was done using randomization codes stored in sealed opaque envelopes in a closed study box within the departments. This type of concealment is less optimal than intervention assignment that is given only following enrollment by central telephone or computer service.

## Conclusions

The findings of this RCT suggest that VMP given in a dosage of 200 mg twice a day following APTL is not an effective treatment to prolong pregnancy or prevent SPTD. The use of VMP was beneficial in twin pregnancies as demonstrated in an exploratory subanalysis and, thus, warrants further investigation.

## Notes

Supplement 1.
Trial Protocol

Supplement 2. **eTable 1.** Characteristics of Singleton Pregnancies **eTable 2.** End Points of Singleton Pregnancies **eTable 3.** Characteristics of Twin Pregnancies

Supplement 3.
Data Sharing Statement
