# Supplementary material for: Vaginal Progesterone for Pregnancy Prolongation After Arrested Preterm Labor: A Randomized Clinical Trial
Source: JAMA Netw Open. 2024 Jul 8;7(7):e2419894. doi: 10.1001/jamanetworkopen.2024.19894 (PMC11231798; doi:10.1001/jamanetworkopen.2024.19894)
Supplement: Supplement 1. — Trial Protocol [file jamanetwopen-e2419894-s001.pdf]

Study protocol

Vaginal progesterone for the prolongation of pregnancy after arrested pre-term labor - multicenter randomized controlled trial, phase 4

Abstract

Background: Preterm labor is the leading cause of neonatal mortality and morbidity. Therefore, once preterm labor is diagnosed before 34 weeks of gestation, tocolytic therapy is initiated in an effort to prolong the pregnancy. Despite meticulous research for useful tocolytics, the medications that are available for this indication have been proven useful for only approximately two days, and searching for a more useful agent for labor arrest is imperative. It has been suggested that progesterone derivatives are useful in preventing preterm labor in women with cervical shortening or preterm labor in the past. However, the impact of progesterone has not been sufficiently tested for pregnancy prolongation after tocolytic therapy.

Working hypothesis and aims: Since progesterone derivatives are useful in preventing preterm labor in cases of risk factors or previous preterm labor, they will also show efficacy in pregnancy prolongation in women whose preterm labor was arrested following tocolytic treatment. The study aim is to evaluate the efficacy of vaginal progesterone for the prolongation of pregnancy after arrested pre-term labor.

Methods: Multicenter randomized controlled trial. Patients diagnosed with arrested pre-term labor following tocolytics at 24-34 gestational weeks will be randomly allocated to receive either vaginal micronized progesterone 400 mg/day (200 mg*2/day) or no treatment. The primary outcomes are the mean number of days from enrollment to delivery and the rate of preterm delivery prior to 37 weeks of gestation. Secondary outcomes include need for additional tocolytic therapy, the rate of pregnancies prolonged for at least one week, neonatal complications, and admission to neonatal intensive care unit.

Assuming mean difference between the groups of 7 days ± 14 days a sample size of 64 women per group will be required (5% two-sided alpha, 80% power). This sample size is sufficient to detect a reduction from 50% to 25% in the rate of women who delivered prior to 37 weeks (5% two-sided alpha, 84% power). Considering 10% drop outs, 140 women will be required for this study (70 per group).

Expected results: Progesterone will be useful to prolong pregnancy after preterm labor was arrested with tocolytics.

Importance: This study has the potential to find a treatment to prevent preterm labor and thus to dramatically reduce neonatal morbidity and mortality.

Probable implications to Medicine: Preterm labor, which is the leading cause for neonatal morbidity and mortality, has major medical, economic, and emotional burdens. Therefore, a possible treatment will be a major medical breakthrough.

Inclusion Criteria

1. 18 years of age

2. Tocolytic treatment between 24+0 and 34+0 weeks

3. Patient's consent to participate in this study

4. 24 hours after tocolytic initiation and up to 3 days after finishing the tocolytic treatment

5. Arrest of preterm labor: 6 or fewer contractions per hour, intact membranes, and ≤ 4cm cervical dilation

Exclusion Criteria

1. Contraindication to ongoing pregnancy including:
    a. Suspected amnionitis during testing for eligibility– evidence of active infection including temperature ≥ 38.0°C and uterine tenderness, foul-smelling vaginal discharge, maternal tachycardia of 120 beats per minute or greater, or sustained fetal tachycardia of 160 beats per minute or greater
    b. Evidence of significant placental abruption (contractions and significant bleeding from placental origin)
    c. Intrauterine fetal death diagnosed at the time of admission
2. Major fetal malformation
3. Known maternal allergy to progesterone
4. Current use of progesterone at the time of admission
5. Epilepsy
6. Breast cancer
7. PPROM (preterm premature rupture of membranes) during testing for eligibility
8. Age below 18 years
9. Known active liver disease (elevated liver enzymes at twice the upper normal limit according to medical history or blood test that were taking doring standard medical care)
10. History of deep vein thrombosis
11. Major active psychiatric disorders (major affective disorders and psychotic disorders)
12. Uncontrolled chronic hypertension

13. Heart failure

14. Chronic renal failure

15. Pre-gestational diabetes with known target organ damage

16. History of spontaneous preterm delivery

17. Previous tocolytic treatment during the current pregnancy

Criteria for patient's removal from the study:

1. Refuse to participate after enrollment

2. No data due to lost to follow-up

3. New allergy to vaginal micronized progesterone

4. An exclusion criteria that was existed before recruitment but was discovered afterwards.

Introduction

Preterm labor is the leading cause of neonatal mortality and morbidity. Worldwide, preterm birth is the most common cause of neonatal death, with about 3.1 million babies per year dying as a direct result of prematurity. Among the survivors, severe morbidity is common and includes infectious and non-infectious respiratory problems, with up to 40% of preterm survivors having bronchopulmonary dysplasia. Additional complications include necrotizing enterocolitis (4-7%), retinopathy (3% after delivery before 28 weeks gestation), hearing impairment (3% in very preterm infants), increased risk for hypoxic ischemic encephalopathy, and intracranial hemorrhage. Cerebral palsy, the most common long-term neurodevelopmental disability, is prevalent in 12% of preterm babies. An additional 19% have motor and co-ordination problems (1). Since prematurity complications could be devastating, once preterm labor is diagnosed before 34 weeks of gestation, a tocolytic therapy is initiated in an effort to prolong the pregnancy (2). Despite meticulous research for useful tocolytics, the medications that are available for this indication have been proven useful only for approximately two days. Since women with arrested preterm labor are at the highest risk for preterm labor, a great deal of effort is invested in the search for a useful agent to prolong their pregnancies.

Previously it has been suggested that progesterone derivatives were useful in preventing preterm labor in women with cervical shortening or preterm labor in the past (3-8). However, the impact of progesterone following tocolytic treatment for pregnancy prolongation should be further investigated. Several small studies with various limitations have shown a potential benefit for progesterone treatment to prolong pregnancy after tocolytic treatment. In the study by Borna et al. (7), they have been able to show efficacy of vaginal progesterone to prolong pregnancy after magnesium sulfate administration for tocolysis. This study was limited by the absence of a placebo group andby the fact that magnesium sulfate is rarely in use today for tocolysis. Oral progesterone also has shown promising results (9), yet the vaginal route of administration should be explored for several reasons. First, the studies that have demonstrated an effectiveness of progesterone in preventing preterm labor after a history of preterm labor used mainly the vaginal route for micronized progesterone (3) and this is the acceptable route for this indication (10). Second, the rationale of the effect of progesterone in preventing preterm labor is by a local effect on the myometrium and probably not by a systemic effect. This will be achieved more easily by vaginal administration. Moreover, in the study by Maher et al. (11) the vaginal progesterone gel was more effective than weekly intramuscular progesterone (systemic route). Finally, since in the vaginal route the progesterone is mainly absorbed locally, the risk for systemic adverse effects and drop out is reduced. The study by Tejada et al., which included 385 women using either 200 mg vaginal micronized progesterone or placebo, did not show a benefit for progesterone (12). Yet, the study methodology had potentially serious flaws; first, over 25% of participating women did not return the unused medication, and among those who did,

the authors stated that they could not be certain that they really administered it. Second, the definition for threatened preterm birth included cervical length ≤30 mm until 31 gestational weeks and/or positive qualitative fetal fibronectin test, which serve more as screening markers for preterm labor than actual signs of threatened preterm birth. Therefore studies with more strict inclusion criteria for preterm birth as well as higher dose of progesterone should be performed. In the present study we would like to investigate the effect of daily 400 mg vaginal micronized progesterone for the prolongation of pregnancy after arrested pre-term labor.

Study hypothesis: Vaginal micronized progesterone will prolong the interval between labor arrest with tocolytic therapy and the development of labor.

Study outcomes

***Primary Outcomes*:**

1. The mean number of days from enrollment to delivery

2. The rate of preterm spontaneous delivery (defined as spontaneous labor or preterm delivery following induction/cesarean section due to preterm premature rupture of membranes prior to 37 weeks of gestation)

***Secondary Outcomes*:**

Obstetrics

1. Number of days from recruitment to repeated preterm labor episode or preterm premature rupture of membranes, up to 37 weeks of gestation
2. Pregnancy prolongation beyond one week
3. Need for repeated acute tocolysis
4. Number of hospitalizations and length of stay until 36.6 gestational weeks
5. The rate of preterm spontaneous labor (defined as spontaneous labor or preterm premature rupture of membranes prior to 37 weeks of gestation)

Neonatal

1. Admission to the NICU (neonatal intensive care unit)
2. Length of NICU stay
3. Length of hospital stay
4. Fetal/neonatal death
5. Birth weight and the rate of small for gestational age neonates
6. The rate of neonatal complications including transient tachypnea, RDS (respiratory distress syndrome), bronchopulmonary dysplasia, ventilatory support, supplemental oxygen, IVH (intraventricular hemorrhage), NEC (necrotizing enterocolitis), PDA (patent ductus arteriosus), retinopathy, neonatal sepsis, and congenital abnormalities not previously identified (specifically genital abnormalities).

Maternal

1. The rate of chorioamnionitis and endometritis
2. Adverse medication reactions (e.g. headache, dizziness)
3. Postpartum hemorrhage
4. Revision of uterine and cervix and reasons for the procedure
5. Urinary tract or vulvovaginal infection until 36.6 weeks

Study population

Women diagnosed with preterm labor that was arrested by tocolytic treatment will comprise the study population. Those women will be recruited from the fetal-maternal medicine unit or delivery unit of Emek Medical Center, Baruch Pade medical center, poriya, Tiberias and Assuta ashdod medical center, Ashdod.

Inclusion Criteria

1. 18 years of age

2. Gestational age at initiation of tocolytic treatment between 24+0 and 34+0 weeks

3. Patient's consent to participate in this study

4. 24 hours after tocolytic initiation and up to 3 days a after finishing the tocolytic treatment

5. Arrest of preterm labor: 6 or fewer contractions per hour, intact membranes, and ≤ 4cm cervical dilation

Exclusion Criteria

1. Contraindication to ongoing pregnancy including:
    d. Suspected amnionitis during testing for eligibility– evidence of active infection including temperature ≥ 38.0°C and uterine tenderness, foul-smelling vaginal discharge, maternal tachycardia of 120 beats per minute or greater, or sustained fetal tachycardia of 160 beats per minute or greater
    e. Evidence of significant placental abruption (contractions and significant bleeding from placental origin)
    f. Intrauterine fetal death diagnosed at the time of admission

2. Major fetal malformation

3. Known maternal allergy to progesterone

4. Current use of progesterone

5. Epilepsy

6. Breast cancer

7. PPROM (preterm premature rupture of membranes) during testing for eligibility

8. Age below 18 years

9. Known active liver disease (elevated liver enzymes at twice the upper normal limit according to medical history or blood test that were taking doring standard medical care)

10. History of deep vein thrombosis

11. Major active psychiatric disorders (major affective disorders and psychotic disorders)

12. Uncontrolled chronic hypertension

13. Heart failure

14. Chronic renal failure

15. Pre-gestational diabetes with known target organ damage

16. History of spontaneous preterm delivery

17. Previous tocolytic treatment during the current pregnancy

Criteria for patient's removal from the study:

1. Refuse to participate after enrollment

2. No data due to lost to follow-up

3. New allergy to vaginal micronized progesterone

4. An exclusion criteria that was existed before recruitment but was discovered afterwards.

Study design: Multicenter open label randomized controlled trial.

Number of estimated medical centers: 3

Screening and enrollment

Women diagnosed with preterm labor arrested by a tocolytic therapy will be evaluated. All women who satisfy the inclusion/exclusion criteria will be offered admission to this study.

Treatments, study groups, and patient management

Patients diagnosed with arrested pre-term labor will be invited to participate and receive vaginal micronized progesterone (Utrogestan– 200mg×2 PV(per vagina) per day) versus no treatment.

Preterm labor will be defined as at least three uterine contractions each lasting 30 seconds or more per 30 minutes confirmed by external tocography and presence of one of the following :

1. Cervical length of 25 mm or less.

2. Cervical dilatation of 1–4 cm accompanied by cervical effacement of at least 50%.

The tocolytic treatment (first, second and third lines) will be determined by the attending physician at the time of admission and later on according to the departmental protocol. In addition, all patients will be screened for GBS (group B *Streptococcus*) colonization if GBS status is unknown and urinary tract infections and treated appropriately. All patients will receive betamethasone for fetal lung maturity and appropriate GBS prophylaxis if those are indicated according to the departmental protocol. The tocolytic treatment which was successful in arresting the preterm labor will be continued for 48 hours. Arrest of preterm labor will be defined as 6 or fewer contractions per hour, intact membranes, and ≤ 4cm cervical dilation.

If the patient has an arrest of preterm labor, she will be asked to participate in this study. She will receive the first dose of the study medication at least 24 hours after initial treatment with tocolytic treatment and up to 3 days after finishing the tocolytic treatment, and then daily until 36+6 weeks or delivery (if occurs before 36.6 weeks).

The cervical length in ultrasonography will be documented prior to patient allocation to study group. Time until delivery will be recorded. In case of preterm premature rupture of membranes (PPROM) progesterone treatment will be discontinued without patient removal from the study.

Ongoing monitoring will include routine obstetric care as well as antepartum testing per the treating physician. Every 3-5 weeks the woman will be invited for follow-up regarding maternal adverse effects and events as well as assessment of the compliance with the study protocol. Final visit/monitoring will be at 36-37 weeks. If the participant prefers not to arrive she will be contacted by phone and will be asked regarding the information mentioned above. Data regarding delivery and neonatal outcomes will be collected from the medical records during delivery and admission in the maternity ward and neonatal/NICU departments. In case of women that will not deliver at the participating medical centers or in case that data will not be collected in the maternity ward, women will be contacted by phone.

Whether the patient should receive repeated courses of tocolytics and/or betamethasone will be left to the discretion of the attending physician according to the departmental protocol.

Data collection

Baseline Characteristics: Age, ethnicity, gravity, parity, gestational age at diagnosis of preterm labor, baseline cervical exam at time of diagnosis, and time of arrest will be collected as mentioned in the CRF.

Information regarding the study outcomes, mentioned above in the study outcome section, will also be collected.

Study randomization

Randomization will be done by computerized software according to power calculation and the codes will be kept inside sealed envelopes. The randomization table will be stratified to 24.0-28.6 and 29.0-34.0 according to the day of tocolytic initiation.

Product description

In this study we will use the progesterone derivative micronized progesterone (Utrogestan 200mg×2 PV per day; BESINS Healthcare), which is approved for use during pregnancy, and was proved to be effective in preventing preterm labor in patients with previous preterm labor and cervical shortening.

The control group will not receive any treatment.

Women who will be allocated to the treatment group will receive a prescription to purchase this medication from the pharmacy. The women will receive a refund for the cost of the medication after presenting the receipt from the pharmacy.

Possible Adverse effects of micronized progesterone (200mg×2 PV per day)

- Changes in appetite and body weight
- Leg edema
- Hyperpigmentation
- Breast congestion
- Nausea
- Dizziness

Severe adverse effects (e.g., anaphylaxis) will be reported to the Helsinki Committee.

Sample Size and statistics

Assuming mean difference between the groups of 7 days ± 14 days a sample size of 64 women per group will be required (5% two-sided alpha, 80% power). This sample size is sufficient to detect a reduction from 50% to 25% in the rate of women who delivered prior to 37 weeks (5% two-sided alpha, 84% power). Considering 10% drop outs, 140 women will be required for this study (70 per group).

Patients' confidentiality

Patients' personal information will be kept in sealed brown envelop separated from the CRF. Each patient will receive a code number written beside the patient name inside the aforementioned envelop. Only the patient's code will be written in the CRF form. All study data will be regarded in a locked closet inside a closed room.

References

1. Platt MJ. Outcomes in preterm infants, Public Health 2014;128:399-403.

2. Carr DB, Clark AL, Kernek K, Spinnato JA. Maintenance oral nifedipine for preterm labor: A randomized clinical trial. American Journal of Obstetrics and Gynecology 1999;181:822-827.

3. da Fonseca EB, Bittar RE, CarvalhoMHB, Zugaib M. Prophylactic administration of progesterone by vaginal suppository to reduce the incidence of spontaneous preterm birth in women at increased risk: A randomized placebo-controlled double-blind study. American Journal of Obstetrics and Gynecology 2003;188(2):419-424.

4. Dodd JM, Flenady V, Cincotta R, Crowther CA. Prenatal administration of progesterone for preventing preterm birth (Review). Cochrane Database System Review 2006:CD004947.

5. Facchinetti F, Paganelli S, Comitini G, Dante G, Volpe A. Cervical length changes during preterm cervical ripening: effects of 17-alpha-hydroxyprogesterone caproate. American Journal of Obstetrics and Gynecology 2007;196(5):453.e1-4.

6. MeisPJ, Klebanoff M, Thom E, et al. Prevention of Recurrent Preterm Delivery by 17 Alpha-Hydroxyprogesterone Caproate. The New England Journal of Medicine 2003;348(24):2379-2395.

7. Borna S, Sahabi N. Progesterone for maintenance tocolytic therapy after threatened preterm labour: A randomized controlled trial. Australian and New Zealand Journal of Obstetrics and Gynaecology 2008:48:58-63.

8. Romero R, Nicolaides K, Conde-Agudelo A, et al. Vaginal progesterone in women with an asymptomatic sonographic short cervix in the midtrimesterdecreases preterm delivery and neonatal morbidity: a systematic review and metaanalysis of individual patient data. Am J Obstet Gynecol 2012;206:124.e1-19.

9. Choudhary M, Suneja A, Vaid NB, Guleria K, Faridi MMA. Maintenance tocolysis with oral micronized progesterone for preventionof preterm birth after arrested preterm labor.International Journal of Gynecology and Obstetrics 2014;126:60-63.

10. ACOG Committee Opinion number 419 October 2008 (replaces no. 291, November 2003). Use of progesterone to reduce preterm birth, Society for Maternal Fetal Medicine Publications Committee. Obstetrics and Gynecology 2008;112(4):963-965.

11. Maher MA, Abdelaziz A, Ellaithy M, Bazeed MF. Prevention of preterm birth: a randomized trial of vaginal compared with intramuscular progesterone. Acta Obstetricia et Gynecologica Scandinavica 2013;92(2):215-222.

12. Martinez de Tejada B1, Karolinski A, Ocampo M, et al. Prevention of preterm delivery with vaginal progesterone in women with preterm labour (4P): randomised double-blind placebo-controlled trial. BJOG 2014 SEP [Epub ahead of print].
