# Supplementary material for: Vaginal Progesterone for Pregnancy Prolongation After Arrested Preterm Labor: A Randomized Clinical Trial
Source: JAMA Netw Open. 2024 Jul 8;7(7):e2419894. doi: 10.1001/jamanetworkopen.2024.19894 (PMC11231798; doi:10.1001/jamanetworkopen.2024.19894)
Supplement: Supplement 2. — eTable 1. Characteristics of Singleton Pregnancies eTable 2. End Points of Singleton Pregnancies eTable 3. Characteristics of Twin Pregnancies [file jamanetwopen-e2419894-s002.pdf]

Supplemental Online Content

Nachum Z, Ganor Paz Y, Massalha M, Wated M, Harel N, Yefet E. Vaginal progesterone for pregnancy prolongation after arrested preterm labor: a randomized clinical trial. *JAMA Netw Open*. 2024;7(7):e2419894. doi:10.1001/jamanetworkopen.2024.19894

eTable 1. Characteristics of Singleton Pregnancies

eTable 2. End Points of Singleton Pregnancies

eTable 3. Characteristics of Twin Pregnancies

This supplemental material has been provided by the authors to give readers additional information about their work.

© 2024 Nachum Z et al. *JAMA Network Open.*

eTable 1. Characteristics of Singleton Pregnancies

	VMP N=53	No treatment N=49	P value
Maternal age (years)	26.6 (5)	26.8 (4.7)	0.70
BMI before pregnancy (Kg/M^2)	23 (4)	23.1 (4.9)	0.84
Number of previous deliveries	0.6 (1)	0.6 (1.1)	0.97
Number of previous early miscarriages	0.2 (0.7)	0.2 (0.6)	0.89
Number of previous late miscarriages	0 (0)	0 (0)	1
Urinary tract infection during pregnancy	11 (21%)	5 (10%)	0.14
Pre-gestational diabetes	1 (2%)	0 (0%)	1
Gestational diabetes	2 (4%)	4 (8%)	0.42
Gestational week of tocolytic treatment	31.7 (2.2)	31.4 (2)	0.20
Tocolytic medication: Nifedipine	53 (100%)	49 (100%)	-
Atosiban	20 (38%)	13 (27%)	0.23
Indomethacin	1 (2%)	2 (4%)	0.61
Gestational week at recruitment	32.0 (2.2)	31.7 (2)	0.25
Cervical length before tocolytic treatment (mm)	18 (6)	19 (6)	0.76
Cervical length before recruitment (mm)	21 (9)	23 (9)	0.20
Cervical dilatation before tocolytic treatment (cm)	1.2 (0.8)	1.2 (0.7)	0.68
Cervical effacement before tocolytic treatment	70% (20%)	60% (20%)	0.27
Presenting part station before tocolytic treatment	-2.4 (0.6)	-2.3 (0.6)	0.37
Cervical dilatation before recruitment (cm)	0.8 (0.6)	0.9 (0.8)	0.49
Cervical effacement before recruitment	50% (20%)	50% (30%)	0.73
Presenting part station before recruitment	-2.6 (0.5)	-2.3 (0.7)	0.13

Values are presented as mean (SD) or number (percent)

Missing: No treatment group: BMI-1, Cervical length before tocolytic treatment-19, Cervical length before recruitment -4, Cervical effacement before tocolytic treatment - 1, Cervical effacement before recruitment -1.

VMP group: Cervical length before tocolytic treatment - 24, Cervical length before recruitment -1

Please note that cervical length was used to evaluated preterm labor only in closed cervix and therefore this measurement was not mandatory in all women.

Abbreviations: BMI, body mass index; VMP, vaginal micronized progesterone.

© 2024 Nachum Z et al. *JAMA Network Open.*

eTable 2. End Points of Singleton Pregnancies

	VMP N=53	No treatment N=49	P value
**Delivery week**	37.6 (3)	37.6 (2)	0.23
**Pregnancy prolongation from recruitment (days)**	39.1 (17.8)	40.8 (20.6)	0.65
**Pregnancy prolongation until 37 weeks (days)**	28.4 (12.9)	32.8 (16.8)	0.14
**Pregnancy prolongation beyond 7 days**	50 (94%)	46 (94%)	1
**Overall preterm delivery**	14 (26%)	16 (33%)	0.49
**Spontaneous preterm delivery**	13 (25%)	13 (27%)	0.82
**Need for additional tocolytic treatment**	3 (6%)	4 (8%)	0.71
**Preterm premature rupture of membranes**	12 (23%)	6 (12%)	0.17
**Number of hospitalizations until 37 weeks**	0.5 (0.7)	0.6 (0.7)	0.67
**length of hospital stay until 37 weeks (days)**	1.4 (2.4)	1.5 (1.9)	0.57
**Number of preterm labor events**	0.2 (0.4)	0.2 (0.4)	0.52
**Number of UTI events until 37 weeks**	0.2 (0.5)	0.4 (0.9)	0.41
**Number of VVI events until 37 weeks**	0 (0.1)	0 (0.1)	0.97
**Delivery mode: Vaginal**	45 (85%)	41 (84%)	0.26
**Vacuum extraction delivery**	1 (2%)	4 (8%)	
**Cesarean delivery**	7 (13%)	4 (8%)	
**Labor induction**	12 (23%)	12 (24%)	0.83
**Chorioamnionitis***	6 (11%)	2 (4%)	0.27
**Endometritis**	1 (2%)	0 (0%)	1
**Postpartum hemorrhage**	6 (11%)	2 (4%)	0.27
**Manual exploration of the uterine cavity and cervix**	5 (9%)	1 (2%)	0.21
**Neonatal gender: female**	18 (34%)	13 (27%)	0.41
**Birth weight (gr)**	2906 (654)	2997 (481)	0.95
**SGA**	2 (4%)	2 (4%)	1
**Apgar score at 1 minute**	9.2 (0.7)	8.9 (1.2)	0.34
**Apgar score at 5 minutes**	9.9 (0.4)	9.8 (0.5)	0.46
**Cord pH**	7.3 (0.1)	7.3 (0.1)	0.09
**Length of hospital stay**	6.7 (12.4)	4.8 (4.6)	0.77
**NICU admission**	8 (15%)	6 (12%)	0.68
**Length of NICU stay**	5.1 (14.6)	1.8 (5.4)	0.57
**Transient tachypnea**	5 (9%)	0 (0%)	0.06
**Respiratory distress syndrome**	1 (2%)	1 (2%)	1
**Chronic lung disease**	1 (2%)	0 (0%)	1
**Ventilator support**	6 (11%)	1 (2%)	0.11
**Supplemental oxygen**	6 (11%)	2 (4%)	0.27
**Intraventricular hemorrhage**	0	0	
**Patent ductus arteriosus**	1 (2%)	0 (0%)	1
**Retinopathy**	1 (2%)	0 (0%)	1
**neonatal sepsis**	1 (2%)	0 (0%)	1

Values are presented as mean (SD) or number (percent)

* Four and two women in the micronized progesterone and no treatment groups, respectively, had subclinical positive placental/membranes growth in culture. The rest had clinical chorioamnionitis.

Abbreviations: UTI, urinary tract infection; VMP, vaginal micronized progesterone ; VVI, vulvovaginal infection

© 2024 Nachum Z et al. *JAMA Network Open.*

eTable 3. Characteristics of Twin Pregnancies

	VMP N=12	No treatment N=15	P value
**Maternal age (years)**	30.6 (4.0)	31.4 (5.1)	0.39
**BMI before pregnancy (Kg/M^2)**	24.4 (3.4)	26.2 (4.8)	0.5
**Number of previous deliveries**	1.1 (1.0)	1.6 (1.6)	0.56
**Number of previous early miscarriages**	0.6 (0.8)	0.3 (0.6)	0.4
**Number of previous late miscarriages**	0 (0)	0 (0)	1
**Urinary tract infection during pregnancy**	0 (0%)	3 (20%)	0.23
**Pre-gestational diabetes**	0	0	-
**Gestational diabetes**	1 (8%)	4 (27%)	0.34
**Gestational week of tocolytic treatment**	30.0 (2.6)	30.6 (2.5)	0.63
**Tocolytic medication: Nifedipine**	12 (100%)	15 (100%)	-
**Atosiban**	5 (42%)	6 (40%)	1
**Indomethacin**	1 (8%)	1 (7%)	1
**Gestational week at recruitment**	30.3 (2.6)	31.0 (2.5)	0.54
**Cervical length before tocolytic treatment (mm)**	19.5 (5.9)	16.2 (3.2)	0.21
**Cervical length before recruitment (mm)**	22.1 (5.0)	18.7 (8.7)	0.06
**Cervical dilatation before tocolytic treatment (cm)**	1.3 (0.6)	1.1 (0.7)	0.42
**Cervical effacement before tocolytic treatment**	70% (10%)	70% (10%)	0.98
**Presenting part station before tocolytic treatment**	-2.5 (0.5)	-2.3 (0.5)	0.41
**Cervical dilatation before recruitment (cm)**	1.1 (1.0)	1.0 (0.9)	0.76
**Cervical effacement before recruitment**	60% (10%)	60% (10%)	0.32
**Presenting part station before recruitment**	-2.5 (0.5)	-2.5 (0.6)	0.88

Values are presented as mean (SD) or number (percent)

Missing: No treatment group: BMI-1, Cervical length before tocolytic treatment-5, Cervical length before recruitment -1.
VMP group: Cervical length before tocolytic treatment - 4, Cervical length before recruitment -2

Please note that cervical length was used to evaluated preterm labor only in closed cervix and therefore this measurement was not mandatory in all women.

Abbreviations: BMI, body mass index; VMP, vaginal micronized progesterone.

© 2024 Nachum Z et al. *JAMA Network Open.*
