# Supplementary material for: Vaginal Progesterone for Pregnancy Prolongation After Arrested Preterm Labor: A Randomized Clinical Trial
Source: JAMA Netw Open. 2024 Jul 8;7(7):e2419894. doi: 10.1001/jamanetworkopen.2024.19894 (PMC11231798; doi:10.1001/jamanetworkopen.2024.19894)
Supplement: Supplement 3. — Data Sharing Statement [file jamanetwopen-e2419894-s003.pdf]

Data Sharing Statement

Nachum. Vaginal Progesterone for Pregnancy Prolongation After Arrested Preterm Labor. *JAMA Netw Open*. Published July 08, 2024. doi:10.1001/jamanetworkopen.2024.19894

Data
Data available: Yes
Data types: Deidentified participant data
How to access data: The data from this study is available from the corresponding author upon a reasonable request and following approval of the institutional review board
When available: With publication

Supporting Documents
Document types: None

Additional Information
Who can access the data: Researchers whose proposed use of the data has been approved
Types of analyses: The data from this study is available from the corresponding author upon a reasonable request and following approval of the institutional review board
Mechanisms of data availability: After approval of a proposal, or with a signed data access agreement
